# Author Correction: The energy cost of polypeptide knot formation and its folding consequences

**DOI:** 10.1038/s41467-017-02384-5

**Published:** 2017-12-14

**Authors:** Andrés Bustamante, Juan Sotelo-Campos, Daniel G. Guerra, Martin Floor, Christian A. M. Wilson, Carlos Bustamante, Mauricio Báez

**Affiliations:** 1 Departamento de Bioquímica y Biología Molecular, Facultad de Ciencias Químicas y Farmacéuticas, Universidad de Chile, Santos Dumont 964, Independencia, Santiago, 8380494 Chile; 2 Departamento Académico de Ciencias Exactas, Facultad de Ciencias y Filosofía, Universidad Peruana Cayetano Heredia, Av. Honorio Delgado 430, San Martin de Porras, Lima-31 15102 Peru; 3 Laboratorio de Moléculas Individuales, Facultad de Ciencias y Filosofía, Universidad Peruana Cayetano Heredia, Av. Honorio Delgado 430, San Martin de Porras, Lima-31 15102 Peru; 4 Department of Molecular and Cell Biology, Department of Physics and Department of Chemistry, Kavli Energy Nanoscience Institute, and Howard Hughes Medical Institute, University of California, Berkeley, CA 94720 USA

Correction to: *Nature Communications* 10.1038/s41467-017-01691-1, pubilshed online 17 November 2017

The original version of this article contained an error in the spelling of the author Christian A.M. Wilson, which was incorrectly given as Christian M.A. Wilson. This has now been corrected in both the PDF and HTML versions of the article.

