# Supplementary material for: The energy cost of polypeptide knot formation and its folding consequences
Source: Nat Commun. 2017 Nov 17;8:1581. doi: 10.1038/s41467-017-01691-1 (PMC5691195; doi:10.1038/s41467-017-01691-1)
Supplement: Supplementary file 1 — Supplementary Information [file 41467_2017_1691_MOESM1_ESM.pdf]

Supplementary Information

Supplementary Note 1

Cluster analysis

In order to cluster the stretched proteins unambiguously, we plotted the average unfolding forces against the average refolding forces for all 32 molecules (Supplementary Fig. 2A). Visual inspection makes evident the presence of two groups: one corresponding to molecules characterized by high average unfolding and refolding force values (26 molecules, turquoise triangles in Supplementary Fig. 2A), the other corresponding to molecules with lower average unfolding and refolding force values (6 molecules, magenta triangles in Supplementary Fig. 2A). Application of an agglomerative cluster analysis (see Material Methods) supports this visual inference. Ninety nine percent of individual events classified as magenta by this analysis, belong to molecules whose average unfolding and refolding forces are low. Similarly, 97% of the events classified as turquoise correspond to molecules whose average unfolding and refolding forces are high. The exception is molecule number 15 (enclosed into a square in Supplementary Fig. 2A). The cluster analysis classified it into the magenta group although visual inspection suggests it belongs to the turquoise group. This happens because the events of molecule 15 are closer to the magenta group center than to the turquoise group center.

Supplementary Methods

Monte Carlo simulations of open polymer chains of finite diameter

The entropic free energy cost of forming a trefoil knot in an open polymer chain was calculated through a Monte Carlo (MC) method developed by Klenin and co-authors[1]. The MC method was applied to explore the conformational space of a self-avoiding polygonal chain as function of the chain diameter. For each diameter, the conformational space was interrogated to determine the frequency and type of knots. This information was used to obtain the entropic energy cost to form a 3_1 knot together with the radius of gyration of the resulting knotted chain as a function of its diameter. A validation of the code is also presented.

Model. We modeled the open chains as self-avoiding polygonal chains (SAPC) represented by N impenetrable right cylindrical segments of unit length and diameter d: The cylinders' axes are represented by unit vectors $\mathbf{e_1}, \dots, \mathbf{e_N}$ and the vertices between cylinders are given by $v_0 = 0, v_k = \sum_{i=1}^{k} e_i, k = 1, \dots, N$. The excluded volume (EV) effect is enforced by requiring that the distance between the axes of non-adjacent cylinders be greater than d. As a result, non-adjacent cylindrical segments do not overlap whereas adjacent segments may overlap (thus yielding a self-avoiding freely jointed chain). We used a SAPC with cylindrical segments of arbitrary length $\ell > 0$ and diameter D by taking ℓ as the unit of length and defining d as the relative chain diameter $d = D/\ell$.

Generation of the conformational ensemble of chains. An ensemble of SAPCs was

obtained using the "hedgehog" method[1]. First, a set of N vectors $\mathbf{e_i}$–the axes of the cylinders– is sampled uniformly at random from the unit sphere (a "hedgehog" as the vectors have a common origin). Next, for each vector $\mathbf{e_i}$ in the set, a non-parallel vector $\mathbf{e_j}$ is randomly chosen, with the pair then being rotated by a random angle ϕ about the axis determined by $\mathbf{e_i} + \mathbf{e_j}$,. This process is repeated about 500 times. The vectors of this random hedgehog are then randomly selected to build a chain. Last, if $d > 0$, this chain is checked for compliance with the EV constraint: if the distance between the axes of any pair of non-adjacent cylinders is greater than d, then the chain is accepted, otherwise it is discarded.

Identification of knotted chains present in the ensemble of open chains. The knot type of a particular chain is determined as follows. Using Taylor's KNOT program[2], the chain was iteratively contracted around its fixed termini as if it were a rubber band, checking that not any two parts of it passed through each other. If within 500 iterations the chain is reduced to just its termini, it is classified as unknotted. Otherwise, the resulting configuration is fed into the Harris and Harvey's Knot program[3], which computes the Alexander polynomial of the configuration to determine its knot type (this program implements the algorithm developed by Vologodskii and co-workers[4]). In what follows we shall denote by $\mathcal{K}$ the set of all knots with 10 or fewer crossings[5]. The knot with 3 crossings is called the trefoil or 3_1 knot, and that with 0 crossings is called the trivial knot, unknot or 0_1 knot.

Calculation of the knotting probability. The probability of formation of an N-segment 3_1 knot of diameter d is obtained as

$$P_{3_1}(N,d) = \frac{n_{3_1}(N,d)}{n(N,d)}, \qquad (1)$$

where $n(N,d)$ is the number of N-segment cylindrical SAPCs of diameter d in the sample of accepted configurations in the experiment, out of which $n_{3_1}(N,d)$ have a 3_1 knot.
From $n_{\exp}$ such experiments, yielding knotting probabilities $P_{3_1,l}(N,d), 1 \leq l \leq n_{esp}$, the best estimate of the probability $P_{3_1}(N,d)$ is obtained as:

$$P_{3_1}(N,d) \approx \hat{P}_{3_1}(N,d) \pm \frac{\hat{S}_{3_1}(N,d)}{\sqrt{n_{\exp}}} \qquad (2)$$

where

$\hat{P}_{3_1}(N,d) = \frac{1}{n_{exp}} \sum_{l=1}^{n_{exp}} P_{3_1,l}(N,d)$ and

$$\hat{S}_{3_1}^2(N,d) = \frac{1}{n_{\exp} - 1} \sum_{l=1}^{n_{\exp}} (P_{3_1,l}(N,d) - \hat{P}_{3_1}(N,d))^2, \qquad (3)$$

are the sample mean and variance, respectively. Here, the probabilities $P_{3_1,l}(N,d)$ are assumed to be independent and identically distributed with mean θ and variance v that are unknown, so that[6] θ is estimated with the unbiased estimator $\hat{P}_{3_1}(N,d)$ and v with the

unbiased estimator $\hat{S}^2_{3_1}(N,d)$. In particular, the variance (mean squared error) v/n_{exp} of the sample mean in (Eq. 2) is estimated by $\hat{S}^2_{3_1}(N,d)/n_{\text{exp}}$.

Previous studies with closed DNA modeled as a freely-jointed chain of cylindrical segments, found an exponential decay relation of the probability of knotting with the segment's diameter-to-length ratio[1]. Here, Arc-L1-Arc is modeled as an open freely-jointed chain with $N = 30$ Kuhn segments of diameter D and length $2P$, where P is the persistence length of the polypeptide. In the notation of our model and in Kuhn length units, the polypeptide is represented by an SAPC of $N = 30$ cylindrical segments of length one and diameter $d = D/2P$ (the segment's diameter-to-length ratio).

In the simulations with $N = 30$, samples of $n(30,d) = 10^6$ accepted configurations are taken for each value of d from 0.00 to 0.40 in increments of 0.05. In all, 60 simulations are done for $d = 0.00$; 70 for $d = 0.20$ and 0.40; and 50 for $d = 0.05, 0.10, 0.15, 0.25, 0.30$ and 0.35.

The estimated knotting probabilities $P_{3_1}(N,d)$ are then fitted with the relation

$$P_{3_1}(N,d) = \alpha_{3_1} exp\left(-\beta_{3_1} d\right) \qquad (4)$$

where $\alpha_{3_1} = 0.024 \pm 0.002$ and $\beta_{3_1} = 18.3 \pm 0.6$.

Calculation of the topological entropic energy. The topological entropy[7] of an N-segment SAPC of diameter d with a K-knot is given by $S_K(N,d) = k_B \ln n_K(N,d)$. Likewise, we have for an SAPC lacking any topological constraint, $S(N,d) = k_B \ln n(N,d)$, where $n(N,d) = \sum_{K\in\mathcal{K}} n_K(N,d)$. The decrease in entropy of the SAPC due to keeping a K knot is therefore $S(N,d) - S_K(N,d) = -k_B \ln P_K(N,d)$. Writing $G_S(N,d) = -TS(N,d)$ and $G_{S_K}(N,d) = -TS_K(N,d)$ for the entropic contributions to the free energies of topologically unconstrained and K-knotted SPACs, respectively, we get

$$\Delta G_{S_{3_1}}(N,d) = G_{S_{3_1}}(N,d) - G_S(N,d) = -k_B T \ln P_{3_1}(N,d), \quad (5)$$
$$\Delta G_{S_{0_1}}(N,d) = G_{S_{0_1}}(N,d) - G_S(N,d) = -k_B T \ln P_{0_1}(N,d), \quad (6)$$

so that the free energy cost of forming the 3_1-knot is

$$\Delta G_{S_{3_1,0_1}}(N,d) = \Delta G_{S_{3_1}}(N,d) - \Delta G_{S_{0_1}}(N,d) = -k_B T \ln\left(\frac{P_{3_1}(N,d)}{P_{0_1}(N,d)}\right). \quad (7)$$

In Fig. 4B of the main text we show $\Delta G_{S_{3_1,0_1}}$ as a function of thickness d for a trefoil knot of length $N = 30$ and thickness d in the range 0.00 to 0.40. Taking the experimentally-determined values of the diameter (0.58 nm) and persistence length (0.7 nm) for a polypeptide chain, we obtain a thickness of $d = 0.4$. For this value, the observed difference

in free energy between the unknotted and 3_1-knotted chain states is about 6.4 kcal mol^{-1}, in agreement with the value deduced from the experimental data, 5.8 ± 1 kcal mol^{-1}.

In order to obtain the variation of the free energy cost of forming a 3_1 knot and all-knots with 10 or fewer crossings ($\mathcal{K}$) as a function of the chain length (Figure 4C), we calculated their probability of occurrence in N-segment SAPCs of $N = 15, 20, 30, 40$ and 100 for a fixed thickness of $d = 0.35$ (or effective diameter of 0.49 nm). For $N = 15, 20, 30$ and 40, calculations were performed over 60×10^6 accepted configurations each, whereas for $N = 100$ calculations were done over 924×10^4 accepted configurations. (The number of configurations above has been given as number of simulations $\times$ accepted configurations per simulation.). In this range of values of N, the free energy cost of forming a 3_1 knot and all-knots were fitted with an exponential decay function plus an offset constant yo,

$$\Delta G_s = yo + \alpha \exp(-\beta N), \qquad (8)$$

where $yo = 4.4 \pm 0.07$ kcal mol^{-1}, $\alpha = 4 \pm 0.14$ kcal mol^{-1} and $\beta = 0.033 \pm 0.026$. Within the accuracy of the calculations, the fitted curve for all-knots in $\mathcal{K}$ was indistinguishable from that for the 3_1 knot.

Knotting Probability for a ring polymer. A closed chain is modeled by a self-avoiding polygon (SAP), which is a SAPC whose ends coincide. This introduces a closure constraint requiring that the sequence of cylinders' axes forms a polygon. The conformational ensemble of SAPs is generated as for the SAPCs save for the following change made to enforce the closure constraint[1]: We choose an even number, N, of vectors $\mathbf{e_i}$–the cylinders' axes–so that the odd-indexed vectors are chosen randomly and independently, whereas the even-indexed ones are set $\mathbf{e_i} = -\mathbf{e_{i-1}}$. Thus, $\sum_{i=1}^{N} \mathbf{e_i} = 0$, which naturally enforces the closure constraint. All subsequent operations carried out to exclude pair correlations between the vectors always leave this sum invariant. The resulting chain is automatically closed.

Supplementary Fig. 7A shows the probability of being knotted for closed chains (SAP) and open chains (SAPC) of length $N = 30$ as a function of the cylinder's diameter d. For SAPs, samples of $n(30, d) = 10^5$ accepted configurations are taken for each value of d from 0.00 to 0.12 by 0.02. In all, 40 simulations (4 sets of 10 simulations each) are done for each d. For SAPCs, samples of $n(30, d) = 10^6$ accepted configurations are taken as specified earlier in the section of the calculation of the knotting probability. Our numerical estimates of the probabilities for the SAPs yield almost identical results to those of Klenin and coworkers[1] and Shimamura and Deguchi[8], thus validating our implementation of the hedgehog algorithm. When fitted with an exponential decay empirical equation, Eq. (4), these probabilities yield a proportionality constant, $\beta_{knot} = 21.9 \pm 0.5$) (Supplementary Fig. 7B), a value lower than that reported by Klenin and coworkers[1] ($\beta_{knot} = 27 \pm 2$). This latter value, however, was calculated averaging probabilities of different chains lengths (14 to 30 segments). The proportionality constant computed by us for closed 3_1 knots, $\beta_{3_1} = 20.0 \pm 0.3$, is identical to the proportionality constant, β_{3_1}, determined for open chains, and is comparable to that reported by Rybenko and coworkers[9] ($\beta_{3_1} = 22$), obtained from the analysis of closed wormlike chains of 16 to 60 segments. However, when the fitting of the

open chains' data to an exponential decay function is extended to thickness d of 0.4 the proportionality constant decreases to β_{3_1} = 18.3. This occurs because the variation of the observed probabilities with chain thickness depart from an exponential function as is apparent from the χ^2/DF obtained for an exponential fitting (χ^2/DF = 5.0). Such deviation for chains of 30 segments is also observed in the work of Klenin and coworkers[1] without interpretation.

Calculation of the radius of gyration. The square radius of gyration of an N-segment SAPC of diameter d is given by

$$R_G^2(N,d) = \frac{1}{(N+1)^2}\sum_{i=0}^{N-1}\sum_{j=i+1}^{N} d_{ij}^2. \qquad (9)$$

where d_{ij} is the distance between the SAPC's vertices v_i and v_j. The mean-square radius of gyration of such chains is then

$$\langle R_G^2\rangle(N,d) = \frac{1}{(N+1)^2}\left\langle\sum_{i=0}^{N-1}\sum_{j=i+1}^{N} d_{ij}^2\right\rangle, \qquad (10)$$

where the brackets $\langle\cdots\rangle$ denote the average over all possible configurations of $\{v_0,\dots,v_N\}$. The root-mean-square radius of gyration is denoted by $R_G(N,d) = \sqrt{\langle R_G^2\rangle(N,d)}$.

In an experiment, where out of a sample of $n(N,d)$ chains $n_K(N,d) > 0$ are K-knotted, the mean-square radii of gyration of K-knotted and of all chains are computed respectively as

$$\overline{R_{G_K}^2}(N,d) = \frac{\sum_{i_K=1}^{n_K(N,d)} R_{G,i_K}^2(N,d)}{n_K(N,d)}, \qquad (11)$$

$$\overline{R_G^2}(N,d) = \frac{\sum_{K\in\mathcal{K}} n_K(N,d)\overline{R_{G_K}^2}(N,d)}{\sum_{K\in\mathcal{K}} n_K(N,d)}. \qquad (12)$$

As for the probabilities, if the experiment is repeated n_{exp} times, yielding root-mean-square radii of gyration $\bar{R}_{g,j}(N,d), 1 \le j \le n_{exp}, g \in \{G_K, G\}$, the best estimate for the root-mean-square radius of gyration $R_g(N,d)$is

$$\bar{R}_g(N,d) = \hat{R}_g(N,d) \pm \frac{\hat{S}_g(N,d)}{\sqrt{n_{\mathrm{exp}}}}, \qquad (13)$$

where $\hat{R}_g(N,d)$ and $\hat{S}_g^2(N,d)$ are the sample mean and variance, respectively [see equations (2 and 3)]. In the absence of excluded volume interactions, $d = 0.0$, the following well known analytical result holds (assuming unit length segments)

$$\langle R_G^2\rangle(N, 0.0) = \frac{N}{6}\frac{N+2}{N+1}, \qquad (14)$$

which can be used to check the accuracy of $\overline{R}_G(N, 0.0)$ obtained from Eq (12).

In Supplementary Fig. 8 we summarize our results for the root-mean-square radius of gyration of SAPCs of length $N = 30$ as a function of their thickness $d = D/2P$ in the range 0.0 to 0.4. When $d = 0.0$ our best estimate of $R_G(30,0.0)$ is $\overline{R}_G(30,0.0) = 2.22 \pm 0.05$. This value is within an error of about 2% from the value, 2.27, obtained from Eq. 14. Furthermore, the trend in the data of the figure suggests that $\overline{R}_G(30, d)$ is a monotonic nondecreasing function of d, leading us to expect that $\bar{R}_G(30,0.4) \approx 2.56 \leq \bar{R}_G(30,0.5)$. Indeed, in their Monte Carlo studies of the freely jointed polymer chain with excluded volume interaction, Baumgärtner and Binder[10] find for chains of length $N = 32$ and excluded volume parameter of 0.5 a root-mean-square radius of gyration of 2.91 ± 0.01. (In their *pearl-necklace* model, a chain consists of $N + 1$ hard spheres of diameter D, connected by N bonds of length $l = 1$, with the excluded volume parameter being given by D/l.)

Validation of the implementation of the hedgehog algorithm. Here, we reproduce some basic results on closed chains of finite width available in the literature using our implementation of the hedgehog algorithm[1,7]. As similar data for open chains are hard to come by in the literature, and as explained in the section below, the hedgehog algorithm for closed and open chains differ only in that in the former a closure condition is enforced, this verification gives us confidence in the use of our implementation of the algorithm for similar calculations with open chains. We have found only one reference[10] giving data (radius of gyration) for open chains with non-zero width based on the perl-necklace model. We have briefly discussed these data in the section of the calculation of the radius of gyration, pointing out, in particular, that our code gives a value within 2% of the well-known analytical value for zero-width chains.

Supplementary figures and tables

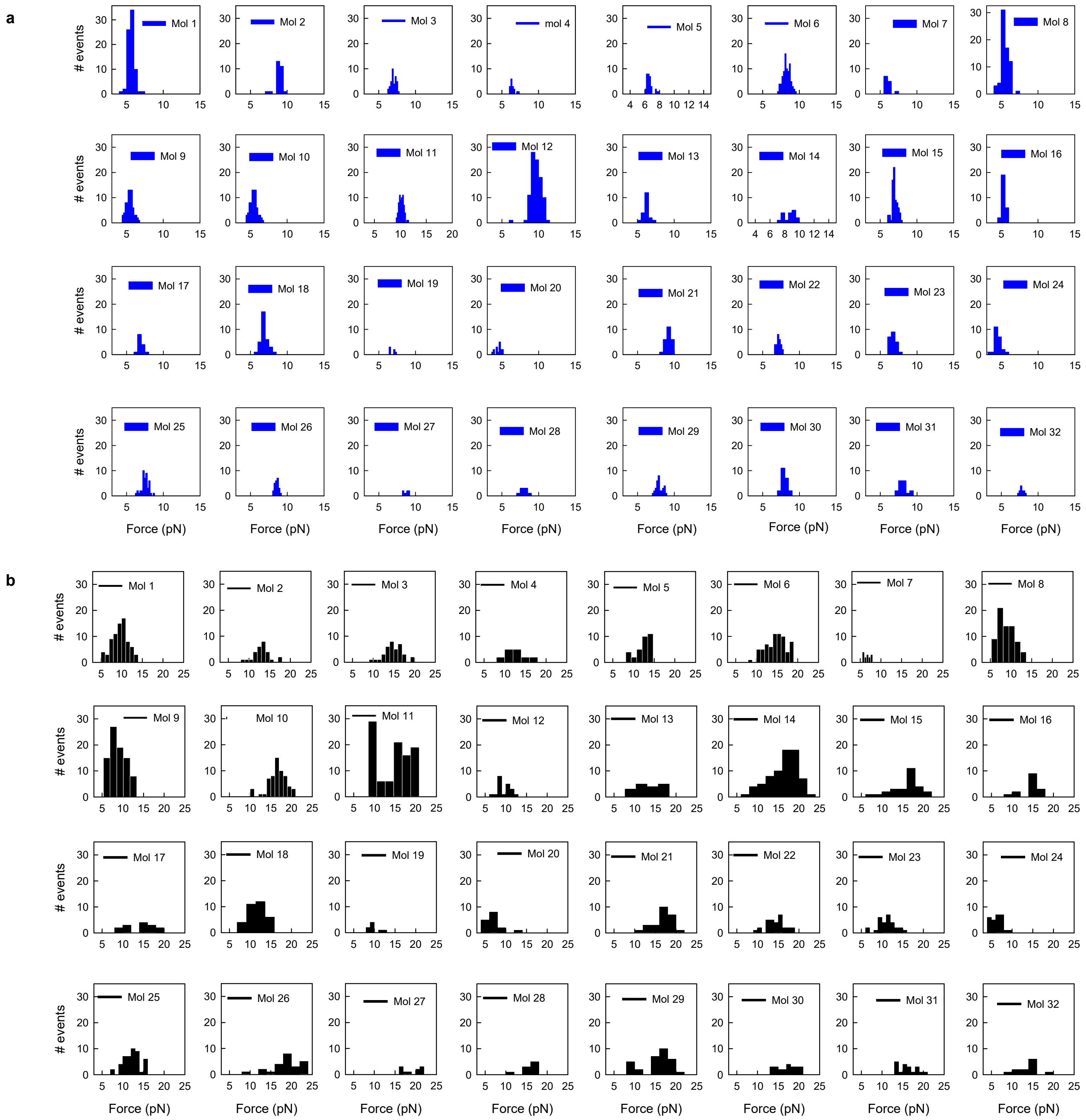

Supplementary Figure 1. Individual force histograms for the 32 Arc-L1-Arc molecules. Unfolding (a) and refolding (b) force histogram for each Arc-L1-Arc molecule studied.

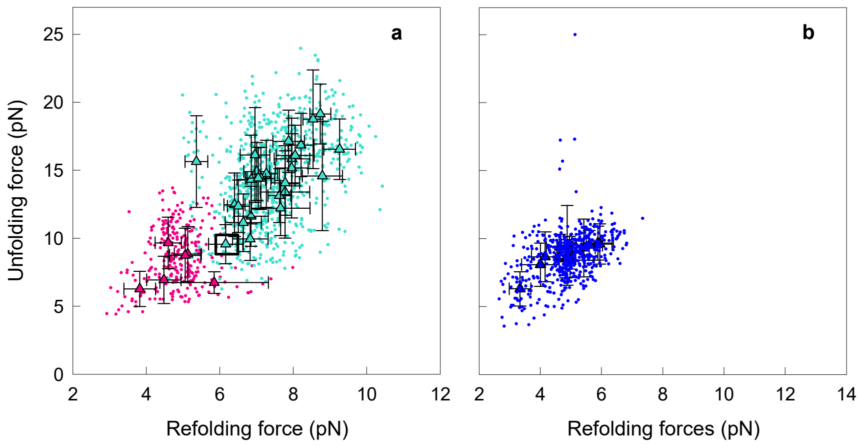

Supplementary Figure 2. Cluster analysis of unfolding and refolding forces of Arc-L1-Arc and pARC. **a.** Each unfolding force is plotted against its corresponding refolding event determined for Arc-L1-Arc (turquoise and magenta dots). Average unfolding and refolding forces obtained for each stretched molecule (turquoise and magenta with errors bars) are also plotted, error bars represent the average SD. Turquoise and magenta symbols represent the Arc-L1-Arc molecules whose unfolding/refolding forces were classified as "high" and "low" respectively. **b.** Cluster analysis of unfolding and refolding forces of pARC. Superimposed on the data are the average unfolding and refolding forces obtained for each stretched molecule (blue triangles with errors bars).

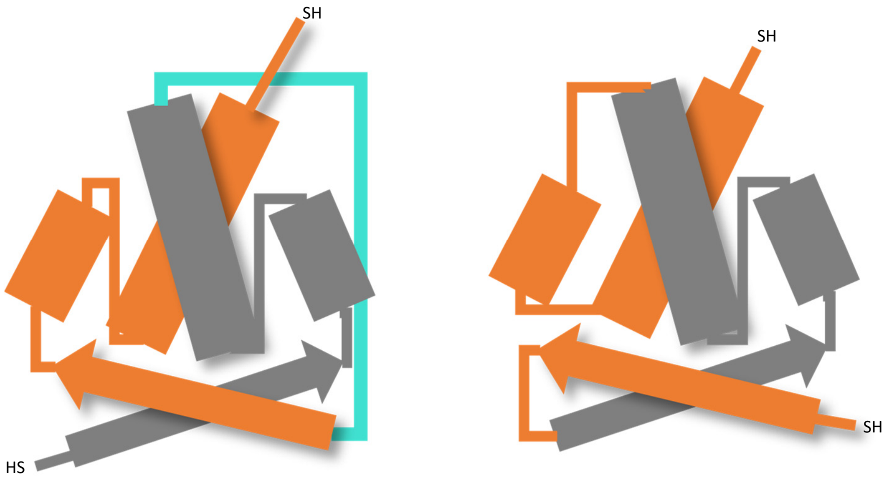

Supplementary Figure 3. Comparison between the topology of Arc-L1-Arc and pARC. Topology of Arc-L1-Arc (left) and pARC (right). In the case of Arc-L1-Arc the loop L1 (turquoise) connect both RHH motifs depicted in orange and gray. The reshuffling of the secondary structure of pARC avoids the linker required to create a monomer with two RHH motifs. The diagram of pARC is based in its X-ray structure (PDB code 1U9P). For the optical tweezers experiments cysteine residues were introduced at positions G3 and G105 of pARC.

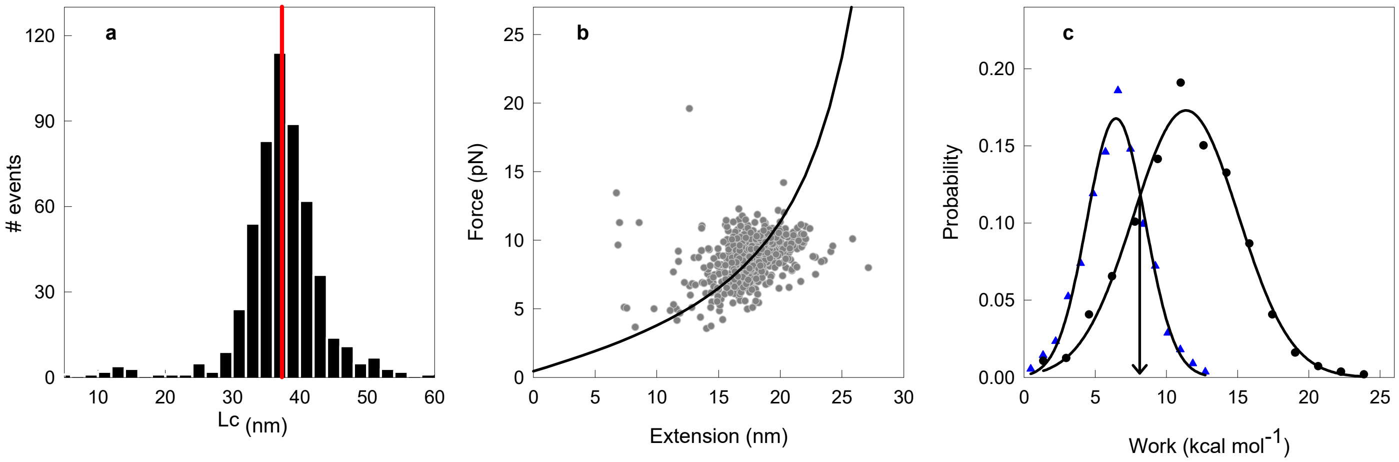

Supplementary Figure 4. Force extensions and work distribution measured for pARC. **a.** The contour length distribution (L_c) for pARC has an average value of 37.1 ± 3.8 nm (red line), in excellent agreement with the expected length of the fully extended protein (36.05 nm). **b.** Force-extension plot for pARC unfolding. The black line represents de WLC force extension curve calculated using an L_c of 37.1 nm and a persistence length of 0.65 nm. **c.** Work distributions for unfolding (●) and refolding (▲) calculated from 1095 unfolding/refolding transitions obtained by stretching 12 molecules of pARC. The arrow indicates the equilibrium work calculated at the intersection point of the two Gaussian curves. ΔG_{pARC} = 8.1 ± 0.1 kcal mol^{-1}, corresponds to the free energy difference between the unfolded (unknotted) and the native (unknotted) state of the protein.

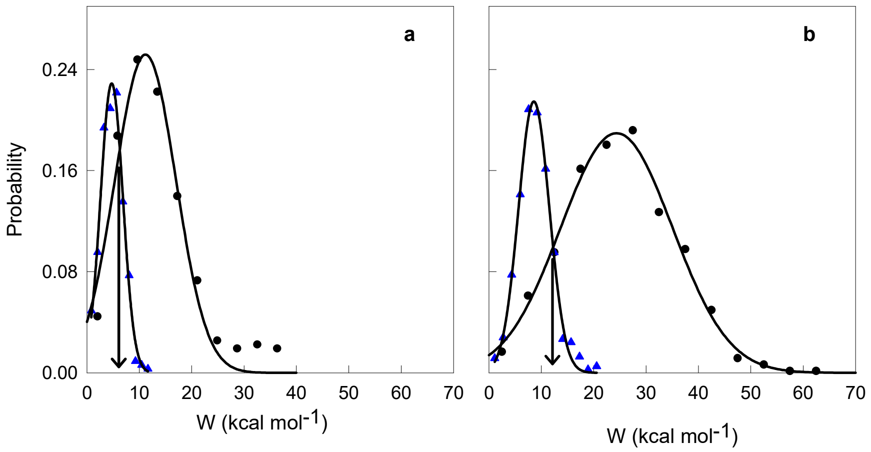

Supplementary Figure 5. Unfolding and refolding work distribution of Arc-L1-Arc. Work distributions of unfolding (●) and refolding (▲) transitions obtained from 6 Arc-L1-Arc molecules (560 transitions) assigned as unknotted **(a)** and 26 molecules (1652 transitions) of Arc-L1-Arc assigned as knotted (**b**). The arrows indicate the equilibrium free energy difference between unknotted-unfolded and unknotted-native states (**a**, $\Delta G_{CFT\ (unknotted)} = 6.2 \pm 0.2$ kcal mol^{-1}) and the knotted-unfolded and knotted-native states (**b**, $\Delta G_{CFT\ (knotted)} = 12.2 \pm 0.2$ kcal mol^{-1}).

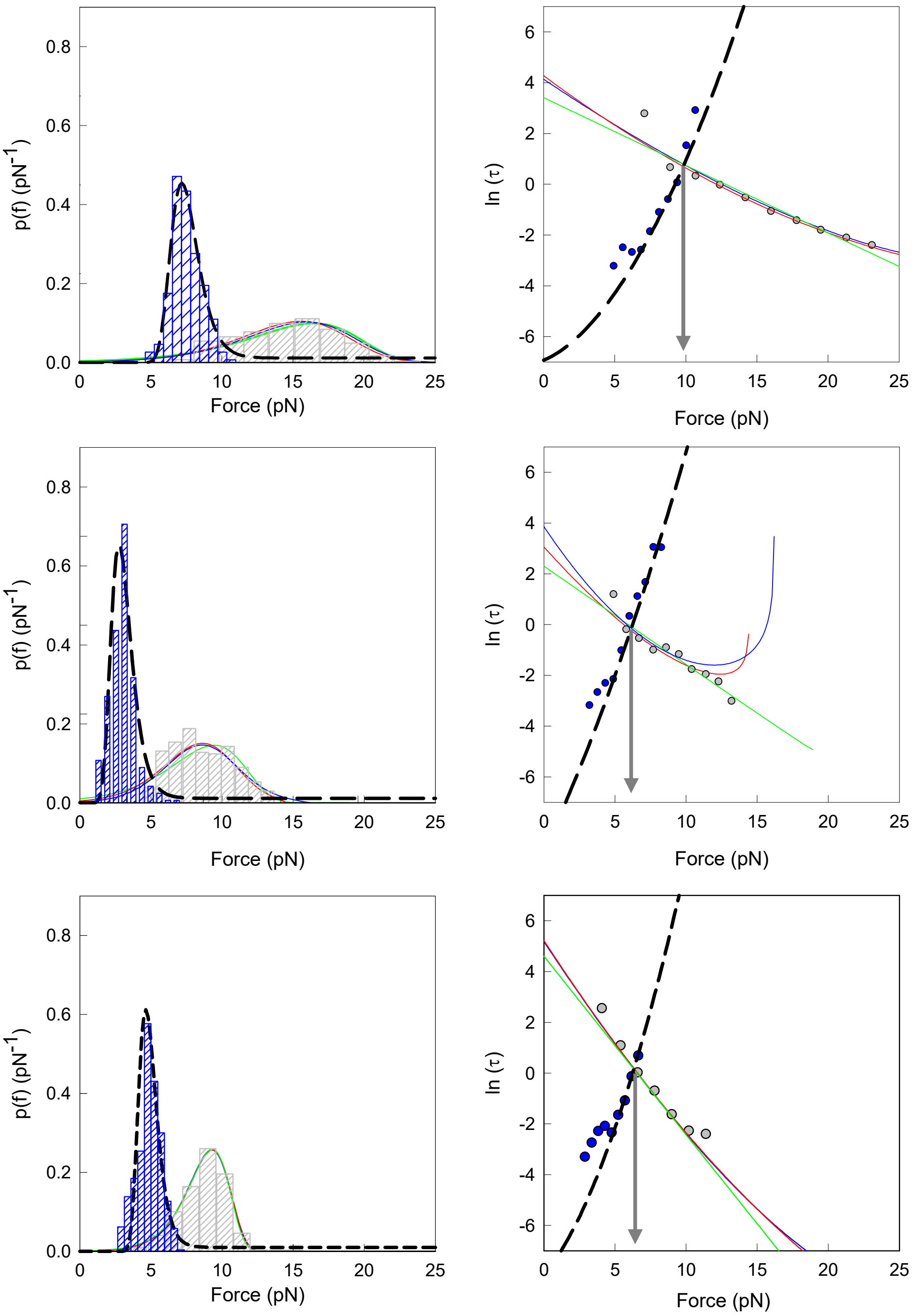

Supplementary Figure 6. Folding and unfolding force distribution for Arc-L1-Arc. Left. Folding (blue bars) and unfolding (gray bars) force distributions for knotted (upper panel), unknotted (middle panel) and pARC (lower panel) molecules classified accordingly to cluster analysis and visual inspection shown in Supplementary Fig. 2 a and b. The right panels represent the semilogarithmic dependence of the lifetimes with the force calculated from the probabilities distribution of folding (blue symbols) and unfolding (gray symbols). The arrow indicates the folding and unfolding lifetimes intersect force at which the molecules spend

half of the time folded or unfolded ($F_{1/2}$), 10 ± 1 pN for knotted Arc-L1-Arc, 6.1 ± 0.5 pN for the unknotted one, and 6.8 ± 0.5 pN for pARC. The molecular extension calculated at $F_{1/2}$ for the knotted, unknotted proteins and pARC are 16.2 ± 1, 13.4 ± 0.9 and 15.4 ± 0.7 nm, respectively. Free energy ($\Delta G_{F1/2}$) is equal ($F_{1/2}$) times the change of extension $\Delta x_{(F1/2)}$ between folded and unfolded states corrected by the stretching work of the unfolded state. The continuous lines represent a fit to Dudko equation to describe the unfolding force distributions (left panels) using parameter υ = 1/2 (blue), υ = 2/3 (red) and υ = 1 (Bell's formula, green) and the semilogarithmic dependence of the unfolding lifetimes (right panels). The dashed lines represent simulations to describe the distribution of refolding forces (left panel) and their semilogarithmic force dependence of lifetimes (right panels) for the parameter υ = ½.

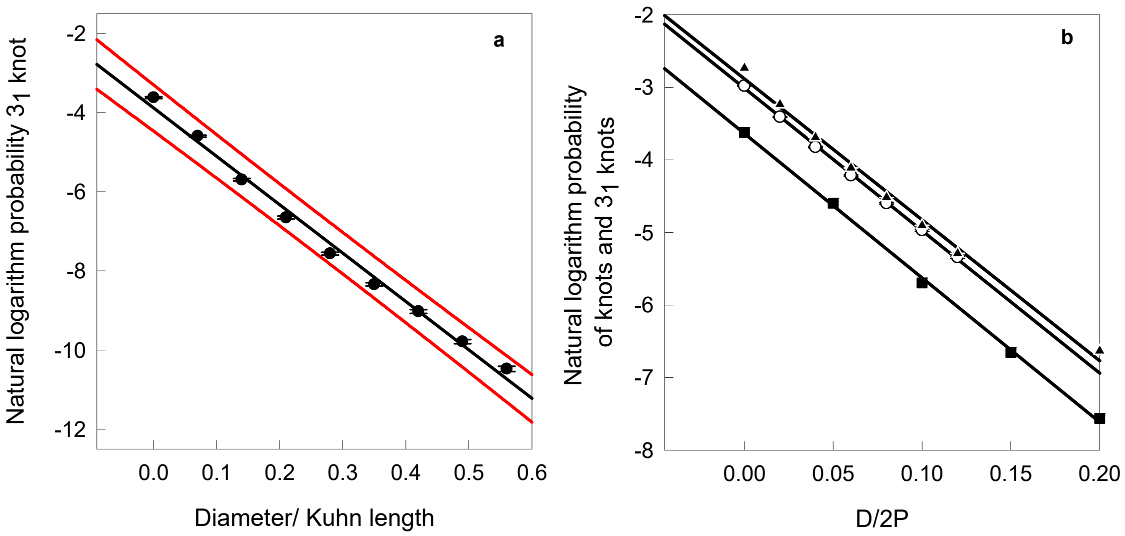

Supplementary Figure 7. **Probability of knot formation in open and closed chains. a.** Semi logarithmic plot of the probability $P_{3_1}(30, d)$ of forming a trefoil (3_1) knot for SAPCs of length $N = 30$ as a function of (scaled) diameter $d = D/2P$. The innermost (central) black curve corresponds to the line of best fit by Eq. (4). The band around this line of best fit (delimited by red curves) is the pointwise 95% prediction band. **b**. Probability of knot formation as a function of chain diameter $d = D/2P$. The probabilities of 3_1 knots in open (closed circle) and closed (open circle) chains are shown alongside the probability of all knots in closed (closed triangles) chains. The thickness of the small bar at each point in the plot is a measure of the error bar in the corresponding computed probability.

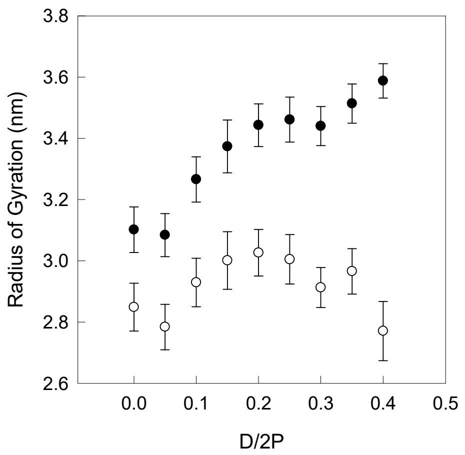

Supplementary Figure 8. Root-mean-square radius of gyration of SAPCs of $N = 30$ segments as a function of their relative diameter, in Kuhn length units (2P, right axis) and for a Kuhn length of 2P=1.4 nm (left axis). Root-mean-square radii of gyration of trefoil ($R_{G_{3_1}}$, open circle) and all (R_G, closed circle) open chains.

**Supplementary Table 1. Kinetic parameters for the unfolding reaction.**					
	Dudko[1]			Bell[2]	
	$^{1}k_u$ (s^{-1})	$^{1}\Delta x_u^{‡}$ (nm)	$^{1}\Delta G_u^{‡}$ (kT)	k_u (s^{-1})	$\Delta x_u^{‡}$ (nm)
ARC-L1-ARC (Unknotted)	0.03 ± 0.1	3.2 ± 2.9	7.3 ± 1.5	0.1 ± 0.05	1.6 ± 0.2
ARC-L1-ARC (knotted)	0.01 ± 0.01	1.8 ± 0.7	9.8 ± 3	0.03 ± 0.008	1.2 ± 0.07
pARC	0.006 ± 0.004	3.7 ± 0.7	17 ± 11	0.01 ± 0.01	2.8 ± 0.3
[1] Average values from υ = 1/2 and 2/3.<br>[2] Values determined for υ = 1					

**Supplementary Table 2. Parameters used to simulate the distribution of folding forces.**					
	Dudko[1]			Bell[2]	
	$^{1}k_f$ (s^{-1})	$^{1}\Delta x_f^{‡}$ (nm)	$^{1}\Delta G_f^{‡}$ (kT)	k_f (s^{-1})	$\Delta x_f^{‡}$ (nm)
ARC-L1-ARC (Unknotted)	10000	6.0	12	90000	9
ARC-L1-ARC (knotted)	1000	2.1	1.0	15000	5
pARC	2000	4.0	5	90000	9
[1] υ=1/2 [2] υ=1					

**Supplementary Table 3. DNA sequences or Arc-L1-Arc mutants**	
Arc-L1-Arc G3C/G121C	ATGAAATGTATGAGCAAAATGCCGCAATTCAATCTGCGTTGGCCGCGTGA<br>AGTCCTGGATCTGGTCCGTAAAGTCGCCGAAGAAAACGGCCGTAGCGTCA<br>ACTCTGAAATTTATCAGCGCGTGATGGAATCTTTTAAAAAAGAAGGCCGT<br>ATCGGTGCGGGCGGTGGCTCAGGTGGCGGTACCGGCGGTGGCTCGGGTGG<br>CGGTATGAAAGGCATGAGCAAAATGCCGCAGTTTAACCTGCGTTGGCCGC<br>GCGAAGTGCTGGATCTGGTGCGTAAAGTTGCCGAAGAAAACGGTCGCAGT<br>GTTAATTCCGAAATTTACCAACGCGTCATGGAATCCTTCAAAAAAGAAGG<br>TCGCATCGGCTGCCATCATCATCATCACCACAAAAACCAACACGAATAA
pArc G3C/G105C	ATGAAATGTATGTCAAAAATGCCGCAATTCAATCTGCGTTGGCCGGGTGG<br>TGGTCCGCAATTCAATCTGCGTTGGCCGCGTGAAGTGCTGGATCTGGTGC<br>GTAAAGTTGCGGAAGAAAACGGTCGCAGCGTTAATTCTGAAATTTATCAG<br>CGTGTCATGGAATCATTTAAAAAAGAAGGCCGCATCGGTGGTACCGGTGG<br>TTCGGGTGGTGGTCGTGAAGTCCTGGACCTGGTCCGCAAAGTGGCCGAAG<br>AAAACGGTCGTAGTGTGAACAGCGAAATTTACCAACGCGTTATGGAATCT<br>TTCAAAAAAGAATGCCGTATCGGTGCCCATCATCATCATCATCACAAAAA<br>CCAACACGAATAA

**Supplementary Table 4. Oligo sequences**	
SH modified oligo	5’-thioMC6-D-GCT-ACC-GTA-ATT-GAG-ACC-AC-3’
Biotinilated oligo	5’-Biotin-CAA-AAA-ACC-CCT-CAA-GAC-CC-3
Digoxigenin modified oligo	5’-Digoxigenin-AA-AAA-ACC-CCT-CAA-GAC-CC-3’

Suplementary References

1. Klenin KV, Vologodskii AV, Anshelevich VV, Dykhne AM, Frankkamenetskii MD. Effect of Excluded Volume on Topological Properties of Circular DNA. *J Biomol Struct Dyn* **5**, 1173-1185 (1988).

2. Taylor WR. A deeply knotted protein structure and how it might fold. *Nature* **406**, 916-919 (2000).

3. Harris BA, Harvey SC. Program for analyzing knots represented by polygonal paths. *J Comput Chem* **20**, 813-818 (1999).

4. Vologodskii A, Lukashin A, Kamenetskii M, Anshelevich V. The knot problem in statistical mechanics of polymer chains. *Sov J Exp Theor Phys* **39**, 1059 (1974).

5. Cha JC, Livingston C. Knotinfo: Table of knot invariants. *preprint*, (2011).

6. Papoulis A, Pillai SU. *Probability, random variables, and stochastic processes*. Tata McGraw-Hill Education (2002).

7. Shimamura MK, Deguchi T. Topological entropy of a stiff ring polymer and its connection to DNA knots. *J Phys Soc Jpn* **70**, 1523-1536 (2001).

8. Shimamura MK, Deguchi T. Gyration radius of a circular polymer under a topological constraint with excluded volume. *Phys Rev E* **64**, (2001).

9. Rybenkov VV, Cozzarelli NR, Vologodskii AV. Probability of DNA knotting and the effective diameter of the DNA double helix. *Proc Natl Acad Sci USA* **90**, 5307-5311 (1993).

10. Baumgartner A, Binder K. Monte-Carlo Studies on the Freely Jointed Polymer-Chain with Excluded Volume Interaction. *J Chem Phys* **71**, 2541-2545 (1979).
